# Nutritional Effects of Removing a Serving of Meat or Poultry from Healthy Dietary Patterns—A Dietary Modeling Study

**DOI:** 10.3390/nu15071717

**Published:** 2023-03-31

**Authors:** Sanjiv Agarwal, Kathryn R. McCullough, Victor L. Fulgoni

**Affiliations:** 1 NutriScience, LLC, East Norriton, PA 19403, USA; 2 Foundation for Meat and Poultry Research and Education, Washington, DC 20036, USA; kmccullough@meatinstitute.org; 3 Nutrition Impact, LLC, Battle Creek, MI 49014, USA; vic3rd@aol.com

**Keywords:** Healthy U.S.-Style Dietary Pattern, Healthy Mediterranean-Style Dietary Pattern, beef, pork, chicken, turkey, cold cut, frankfurter, sausages, bacon, protein, micronutrients

## Abstract

Meat and poultry are nutrient-dense sources of protein and typically are recommended as part of an overall healthy diet. The objective was to assess the nutritional impact of removing a serving of meat/poultry in Healthy Dietary Patterns (HDPs) using a similar approach to that used by the USDA for Dietary Guidelines for Americans. Composites of minimally processed and further processed meat and poultry were developed and their nutrient profiles were used to accomplish modeling by removing nutrients of each meat and poultry composite from the HDPs. The removal of a 3 oz (85 g) serving of meat or poultry resulted in decreases (10% or more from baseline) in protein and several key micronutrients including iron, phosphorus, potassium, zinc, selenium, thiamine, riboflavin, niacin, vitamin B_6_, vitamin B_12_, and choline as well as cholesterol and sodium in the HDPs, and the decreases were consistent for most nutrients with the removal of either minimally processed (fresh) or further processed meat or poultry and even after adjusting for changes in calories. In conclusion, the results of this dietary modeling study show that the removal of a meat and poultry serving from HDPs resulted in decreases in protein and several key nutrients.

## 1. Introduction

Meat, including poultry, is a major component of the US diet and is a predominant source of dietary protein [1]. Dietary Guidelines for Americans (DGA) 2020–2025 [2] and MyPlate [3] recommend the consumption of lean meat and poultry as part of an overall healthy diet. In the U.S., meat comprises a significant portion of the normal diet, contributing more than 15% to daily energy intake, 40% to daily protein intake, and 20% to daily fat intake [4], and over 70% of adults consume red meat or poultry with a mean intake of 14–15 lean oz equivalents (eq)/week [5].

Meat is a dense source of nutrients such as protein, iron, zinc, and B vitamins [6,7]. Animal-sourced protein foods, because of their higher protein quality, are more efficient sources of dietary protein than plant protein foods. Consumption of meat has been criticized from ethical, environmental, and health perspectives in scientific and popular media. However, the meat foods evaluated in these studies as well as the terminology used to describe meat foods in nutrition research has been inconsistent and varies in different studies [8,9]. Meat is generally defined as beef, veal, pork, lamb, and game meat; and poultry is defined as chicken, turkey, Cornish hens, duck, goose, quail, and pheasant (game birds) by USDA [10]. Cured meat (frankfurters, sausages, corned beef, cured ham, and luncheon meat that are made from beef, pork, or poultry) is usually considered a separate food category [10]. While red meat and processed meat have been associated with a variety of chronic diseases in observational studies [11,12,13], minimally processed meat and poultry were not associated with chronic disease risk or related mortality [14,15]. 

To help guide individuals in healthy eating, the USDA developed Healthy Food Patterns and released them as part of DGA 2015–2020 [16] and updated them as Healthy Dietary Patterns for release as part of DGA 2020–2025 [2]. These patterns include the characteristics of healthy eating with details on how to follow the DGA guidance within caloric needs, and these can be used by all individuals for meal planning. Three Healthy Dietary Patterns are developed: (1) The Healthy U.S.-Style Dietary Pattern (USP), which is the primary dietary pattern of the USDA based on food types and the proportions Americans typically consume; (2) The Healthy Mediterranean-Style Dietary Pattern (MSP) which more closely reflects Mediterranean-style diets that are associated with positive health outcomes in studies; and (3) the Healthy Vegetarian Dietary Pattern (VDP) to more closely reflect the eating patterns of vegetarians. These Healthy Dietary Patterns are based on the types and proportions of foods Americans of all ages, genders, races, and ethnicities typically consume, but in nutrient-dense forms, and appropriate amounts and servings of lean meat, poultry, and eggs are included as part of protein foods in USP and MSP. DGA 2020–2025 [2] also suggest that a healthy dietary pattern is associated with beneficial outcomes for all-cause mortality, cardiovascular disease, overweight and obesity, type 2 diabetes, bone health, and certain types of cancer (i.e., breast and colorectal).

However, there is a strong push among scientific advocacy groups and policy makers to limit animal-sourced food products in the diet primarily due to environmental concerns [17,18,19,20,21,22]. Therefore, the aim of this analysis was to examine the potential unintended consequences of limiting meat and poultry by modeling the effect of removing a serving of meat and poultry on nutrient profiles of the healthy dietary patterns identified in the Dietary Guidelines for Americans, 2020–2025, and to assess whether the modeled changes lead to meaningful changes in intake.

## 2. Materials and Methods

To achieve the objective of this study, four different minimally and further processed meat and poultry composites were developed using a total of 397 food codes in 10 food categories [10] using a similar modeling approach as that used by the USDA. The foods were grouped into minimally processed and further processed foods, and further into meat (beef and pork) and poultry (chicken and turkey). These groups are consistent with the meat science classification of meat products [23]. Minimally processed meat and poultry items include raw, uncooked products that have not been significantly altered compositionally and contain no added ingredients, but may have been reduced in size by fabrication, mincing, grinding, and/or a meat recovery system. Further processed meat and poultry items include those that undergo a transformation beyond minimal processing, contain approved ingredients, and may be subject to a preservation or processing step(s) including salting, curing, fermentation, thermal processing (smoking and cooking), batter/breading, or other processes to enhance sensory, quality, and safety attributes. One or two representative food codes were selected in each category, similar to the approach used by the USDA, and proportions of different foods in a category were based on their population-weighted consumptions for NHANES 2017–2018 participants (*n* = 7036; age 2+ years) [24]. The meat composite used in USDA’s Healthy Dietary Patterns [25] was also used as an additional meat option. The following composites were developed and further details are provided in Table 1:

- Meat composite used in USDA’s Healthy Dietary Patterns: USDA meat
- Minimally processed meat: 69.30% Beef; and 30.70% Pork
- Minimally processed poultry: 87.73% Chicken; and 12.27% Turkey.
- Further processed meat: 13.27% Beef; 5.09% Pork; and 81.64% Cold cuts/bacon/frankfurters/sausages
- Further processed poultry: 82.49% Chicken; and 17.51% Cold cuts/bacon/frankfurters/sausages.

The nutrient profile for the meat composite used by the USDA was obtained from the Food Pattern Modeling Report [25]. Nutrient profiles for all representative meat and poultry foods (except for ground beef) were obtained from USDA’s Food and Nutrient Database for dietary Studies (FNDDS) 2017–2018 specific for NHANES 2017–2018 [26]. Nutrient profile for ground beef (FDC ID 173113, Beef, ground, 97% lean meat /3% fat, patty, cooked, pan-broiled) was obtained using USDA Food Data Central [27]. Nutrient profiles for meat and poultry composites were computed by adding the nutrients of component foods in the proportions as described above and are presented in Table 2.

Base nutritional profiles of Heathy Dietary Patterns: USP and MSP for 2000 kcal were obtained from the Food Pattern Modeling Report [25]. Dietary modeling was accomplished by removing nutrients of a 3 oz (85 g) serving of each meat and poultry composite from the Healthy Dietary Patterns (USP and MSP), and modified nutrient profiles were created using Microsoft Excel (Version 2019, Microsoft, Inc., Redmond, WA, USA). Additional modeling approaches were conducted where calories and nutrients were increased from the rest of the diet to match the baseline calories, thus providing an isocaloric removal of meat and poultry servings (i.e., showing the impact of removing meat and poultry and allowing the remaining diet to increase to meet the planned calorie level). To accomplish this, each nutrient value after removal of the meat and poultry composite was multiplied by the baseline calories and divided by the modified calories (Isocaloric nutrient value = {(baseline nutrient value − composite nutrient value) ÷ (baseline calorie value − composite calorie value)} × baseline calorie value). Basically, all the foods in the existing dietary pattern are increased proportionally to the number of calories of meat removed. A change of 10% or more in nutrients due to dietary modeling analyses of Healthy Dietary Patterns was used as an indicator of meaningful differences.

## 3. Results

Removal of a 3 oz (85 g) serving of USDA meat composite from USP resulted in a decrease in protein (−23%), iron (−11%), phosphorus (−12%), zinc (−27%), copper (−11%), selenium (−21%), thiamine (10%), niacin (−21%), vitamin B_6_ (−15%), vitamin B_12_ (−28%), and choline (−22%) (Table 3). Additionally, cholesterol and sodium also decreased (−28% and −18%, respectively) by removing a 3 oz (85 g) serving of meat. However, the decreases for iron, phosphorus, copper, thiamin, and B_6_ were attenuated and became less than 10% from the baseline in the isocaloric scenario (Table 3). Identical results were obtained when a 3 oz (85 g) serving of meat was removed from MSP except that the decrease in thiamin was always less than 10% from the baseline (Table 3).

Removal of a 3 oz (85 g) serving of minimally processed meat from USP resulted in decreases in protein (−27%), iron (−11%), phosphorus (−13%), potassium (−10%), zinc (−30%), selenium (−29%), thiamine (11%), riboflavin (−11%), niacin (−30%), vitamin B_6_ (−25%), vitamin B_12_ (−21%), and choline (−21%) (Table 4). Additionally, cholesterol, saturated fat, and sodium also decreased (−32%, −11%, and −22%, respectively) by removing a 3 oz (85 g) serving of minimally processed meat. However, the decreases for iron, phosphorus, potassium, thiamin, riboflavin, and saturated fat were attenuated and became less than 10% from baseline in the isocaloric scenario (Table 4). Identical results were obtained when a 3 oz (85 g) serving of minimally processed meat was removed from MSP (Table 4).

Removal of a 3 oz (85 g) serving of minimally processed poultry from USP resulted in decreases in protein (−27%), phosphorus (−12%), selenium (−23%), niacin (−37%), vitamin B_6_ (−32%), and choline (−18%) (Table 5). Additionally, cholesterol and sodium also decreased (−38% and −19%, respectively) by removing a 3 oz (85 g) serving of minimally processed poultry. However, the decrease in phosphorus was attenuated and became less than 10% from the baseline in the isocaloric scenario (Table 5). Identical results were obtained when a 3 oz (85 g) serving of minimally processed poultry was removed from MSP (Table 5).

Removal of a 3 oz (85 g) serving of further processed meat from USP resulted in decreases in protein (−20%), MUFA (−15%), phosphorus (−14%), potassium (−11%), zinc (−17%), selenium (−26%), thiamine (−14%), riboflavin (−11%), niacin (−24%), vitamin B_6_ (−13%), B_12_ (−11%), and choline (−19%) (Table 6). Additionally, fat, cholesterol, saturated fat, and sodium also decreased (−12%, −24%, −16%, and −38%, respectively) by removing a 3 oz (85 g) serving of further processed meat. However, the decreases for phosphorus, potassium, zinc (only in USP), thiamin, riboflavin, and vitamins B_6_, B_12_, fat, and saturated fat were attenuated and became less than 10% from baseline in the isocaloric scenario (Table 6). Identical results were obtained when a 3 oz (85 g) serving of further processed meat was removed from MSP except that the decrease in vitamin B_12_ was always less than 10% from baseline (Table 6). 

Removal of a 3 oz (85 g) serving of further processed poultry from USP resulted in decreases in protein (−18%), monounsaturated fatty acids (−14%), polyunsaturated fatty acids (−13%), phosphorus (−12%), selenium (−15%), niacin (−27%), vitamin B_6_ (−15%), and choline (−12%) (Table 7). Additionally, fat, cholesterol, saturated fat, and sodium also decreased (−14%, −26%, −10%, and −28%, respectively) by removing a 3 oz (85 g) serving of further processed poultry. However, the decreases for fat, saturated fat, monounsaturated fatty acids, polyunsaturated fatty acids, phosphorus, selenium, B_6_, and choline were attenuated and became less than 10% from baseline in the isocaloric scenario (Table 7). Generally identical results were obtained when a 3 oz (85 g) serving of further processed poultry was removed from MSP, however, with isocaloric removal of further processed poultry vitamin A and C in USP and vitamin C in MSP also increased by ≥10% from baseline (Table 7).

## 4. Discussion

The results of this dietary modeling analysis show that the removal of a serving of meat or poultry resulted in decreases (10% or more from baseline) in protein and several key micronutrients including iron, phosphorus, potassium, zinc, selenium, thiamine, riboflavin, niacin, vitamin B_6_, vitamin B_12_, and choline as well as cholesterol and sodium in the Healthy Dietary Patterns. It is interesting to note that the decreases were consistent for most nutrients with the removal of either minimally processed or further processed meat or poultry and even after adjusting for the decreases in calories associated with removing meat/poultry servings.

Minimally processed meat used in our study included lean beef steaks and lean pork chops; minimally processed poultry included chicken breasts, drumsticks, and turkey; further processed meat included battered/fried beef steaks, breaded pork chops, spareribs, deli ham, pork bacon, beef hot dogs, and pork sausages; and further processed poultry included grilled and rotisserie chicken breasts, chicken nuggets, deli turkey, turkey bacon, chicken hot dogs, and turkey sausages (see Table 1). Beef is a staple food in the Western diet and is an important source of high-quality protein and several key micronutrients including highly bioavailable iron, zinc, and B vitamins in the American diet [6,7,28,29]. We recently reported that beef also contributes significant amounts of several key micronutrients such as zinc, iron, vitamin B_12_, vitamin B_6_, and choline in the diets of American adults [30]. Pork is one of the most widely consumed meats in the world and accounts for over 30% of global meat production and intake. Pork is a nutrient-rich source of high-quality protein and select nutrients such as potassium, phosphorus, zinc, selenium, thiamin, riboflavin, niacin, and vitamins B_6_ and B_12_ [31,32]. Poultry meat is also high in protein and B-group vitamins (mainly thiamin, vitamin B_6_, and pantothenic acid), and minerals (like iron, zinc, and copper) [33,34].

In the present analysis, removal of a 3 oz (85 g) serving of minimally processed meat from the Healthy Dietary Patterns resulted in ≥10% decreases from baseline in protein, iron, phosphorus, potassium, zinc, selenium, thiamine, riboflavin, niacin, vitamin B_6_, vitamin B_12_, and choline. Similarly, removal of a 3 oz (85 g) serving of minimally processed poultry also resulted in ≥10% decreases in protein, phosphorus, selenium, niacin, vitamin B_6_ and choline. Although there was a consequent small decrease in energy with the removal of meat or poultry from healthy dietary patterns, the decrease was less than 10% from baseline. Interestingly, the decreases in protein, zinc, selenium, niacin, vitamin B_6_, vitamin B_12_, and choline from the removal of meat; and protein, selenium, niacin, vitamin B_6_, and choline from the removal of poultry remained ≥10% from baseline when the decrease in energy was adjusted (isocaloric scenario) by adding back energy/nutrients from the rest of the healthy dietary pattern. This suggests that the meat and poultry are more nutrient-dense foods than other foods in the Healthy Dietary Patterns. Indeed, minimally processed meat or poultry provides about three times more protein, four times more zinc (for meat only), three to four times more selenium, three to four times more niacin, three to four times more vitamin B_6_, and two to three times more choline than Healthy Dietary Patterns on a per 100 kcal basis. However, meat and poultry also provide over four times more cholesterol, ~70% more saturated fat (for meat only), and about three times more sodium.

While lean and fresh/unprocessed meat and poultry are recommended as part of healthy diets [2,3] and are not associated with adverse health outcomes [14,15], intake of processed meat has been reported to be associated with risk for several chronic disease outcomes in scientific research [11,12,13]. On a per 3 oz (85 g) serving basis, further processed meat or poultry provide more calories, less protein and other key micronutrients, and more saturated fat and sodium than their minimally processed counterparts. DGA 2020–2025 has identified saturated fat and sodium as nutrients to limit, as their current intake is more than recommended based on their suspected role in chronic disease outcomes [2]. Additionally, heme iron, *N*-nitroso compounds in processed meat, as well as heterocyclic aromatic amines and polycyclic aromatic hydrocarbons formed during high-temperature processing are also considered, by some, as potential carcinogens in processed meat [35]. However, the removal of further processed meat and poultry such as ground beef, fried steaks, pork chops, spareribs, chicken nuggets, cold cuts, bacon, frankfurters, and sausages, also resulted in ≥10% decreases in protein, selenium, and choline. In a recently published analysis of NHANES 2001–2018, we reported that beef including processed and ground beef contributed to the intake of protein and several key micronutrients [30]. In an earlier analysis of NHANES, intake of lunch meat (deli, cold cuts, or cured meat) did not adversely affect diet quality or physiological parameters in children and adults [36]. Although there is some evidence that high meat consumption (especially red and processed meat) may increase the risk for some types of chronic disease [37], meat (fresh and lean meat) can be an important source of nutrients, especially for people with limited availability of foods.

There has been a consistent ongoing discussion and increasing concerns about the environmental impact of animal-sourced foods and policymakers are increasingly concerned with the environmental consequences of meat consumption in addition to the effect on human health. Some studies show that meat production results in anthropogenic greenhouse gas emissions including CO_2_, methane, and nitrous oxide and is the single most important source of methane [17,18]. Consequently, there has been a strong push to limit or eliminate animal-based foods to minimize environmental impacts [19,20,21,22]. However, such recommendations do not account for their potential effect on food availability and nutrient intake. While removing or limiting animal foods from the diet may help lower greenhouse gas emissions, nutritional inadequacies may occur as potential trade-offs. Thus, recommending limiting animal-sourced foods could have potential unintended consequences [38,39,40]. Our results clearly show that the removal of a serving of meat or poultry could cause decreases in protein and several key nutrients in the Healthy Dietary Patterns.

While we used USDA’s dietary modeling approach for menu modeling of Healthy Dietary Patterns, there are some key aspects to consider when interpreting our results. Firstly, the representative foods for different meat or poultry composites were selected in each category using USDA’s approach, and proportions of different food in a category were based on their population-weighted consumptions using the most recent nationally representative database (NHANES 2017–2018). However, our results are dependent on foods selected in our meat and poultry composites and changes in the items selected for each composite may impact modeling results. Additionally, the results presented here are based on dietary modeling to evaluate the maximum effect of removing meat and/or poultry and may not reflect actual individual dietary behavior; however, such dietary modeling offers a technique to test the potential nutritional impact of dietary guidance. Finally, our results may not apply to non-US cultures as dietary recommendations and current dietary patterns may be different.

## 5. Conclusions

In conclusion, the results of this dietary modeling study show that the removal of a meat and poultry serving from Healthy Dietary Patterns resulted in decreases in protein and several key nutrients associated with meat intake like iron, zinc, and vitamin B_12_ but also phosphorus, potassium, selenium, thiamine, riboflavin, niacin, vitamin B_6_, and choline with considerable consistency in results whether removing minimally processed or further processed meat and poultry. The results also provide insight into the nutritional consequences of removing meat and poultry from Healthy Dietary Patterns and identifies nutrient amounts that may need to be replaced by other foods.

## Figures and Tables

**Table 1:** Composition of meat and poultry composites.

Composites	Proportion (%)
**USDA Meat**	
Meat composite used in USDA’s Healthy Dietary Patterns	100.00
*23.71% Beef*	
*27.12% Beef, ground*	
*12.76% Pork, fresh*	
*6.35% Pork, cured*	
*6.62% Sausage*	
*8.75% Luncheon meats and bacon, beef*	
*12.34% Luncheon meats and bacon, pork*	
*2.35% Others (game meat, lamb and liver)*	
**Minimally processed meat**	
Minimally processed beef (total 38 WWEIA food codes in WWEIA categories 2002 and 2004)	69.30
*83.85% Beef steak, broiled or baked, lean only eaten (WWEIA food code 21101130)*	
*16.15% Beef steak, fried, lean only eaten (WWEIA food code 21102130)*	
Minimally processed pork (total 41 WWEIA food codes in WWEIA category 2006)	30.70
*100% Pork chop, broiled or baked, lean only eaten (WWEIA food code 22101120)*	
**Minimally processed poultry**	
Minimally processed chicken (total 73 WWEIA food codes in WWEIA categories 2202 and 2204)	87.73
*53.25% Chicken breast, grilled without sauce, skin not eaten (WWEIA food code 24123301)*	
*39.06% Chicken breast, baked, broiled, or roasted, skin not eaten, from raw (WWEIA food code 24122131)*	
*7.69% Chicken drumstick, sauteed, skin not eaten (WWEIA food code 24144301)*	
Minimally processed turkey (total 26 WWEIA food codes in WWEIA category 2206)	12.27
*100% Turkey, light meat, roasted, skin not eaten (WWEIA food code 24201120)*	
**Further processed meat**	
Further processed Beef (total 10 WWEIA food codes in WWEIA categories 2002 and 2004)	13.27
*20.90% Beef steak, battered, fried, NS as to fat eaten (WWEIA food code 21104110)*	
*79.10% Ground beef patty, cooked (FDC ID 173113)*	
Further processed pork (total 17 WWEIA food codes in WWEIA category 2006)	5.09
*75.05% Pork chop, breaded or floured, broiled or baked, lean only eaten (WWEIA food code 22101150)*	
*24.95% Pork, spareribs, barbecued, with sauce, lean only eaten (WWEIA food code 22701050)*	
Cold cuts, bacon, frankfurters & sausages (total 84 WWEIA food codes in WWEIA categories 2602, 2604, 2606 and 2608)	81.64
*46.29% Ham, prepackaged or deli, luncheon meat, reduced sodium (WWEIA food code 25230220)*	
*18.05% Pork bacon, NS as to fresh, smoked or cured, reduced sodium, cooked (WWEIA food code 22600210)*	
*27.27% Frankfurter or hot dog, beef, reduced fat or light (WWEIA food code 25210620)*	
*8.39% Pork sausage, reduced sodium (WWEIA food code 25221408)*	
**Further processed poultry**	
Further processed Chicken/Turkey (total 91 WWEIA food codes in WWEIA categories 2202, 2204 and 2206)	82.49
*34.54% Chicken breast, grilled with sauce, skin not eaten (WWEIA food code 24123311)*	
*15.27% Chicken breast, rotisserie, skin not eaten (WWEIA food code 24122171)*	
*50.18% Chicken nuggets, from fast food (WWEIA food code 24198731)*	
Cold cuts, bacon frankfurters & sausages (total 15 WWEIA food codes in WWEIA categories 2602, 2604, 2606 and 2608)	17.51
*80.11% Turkey, prepackaged or deli, luncheon meat, reduced sodium (WWEIA food code 25230785)*	
*8.30% Turkey bacon, reduced sodium, cooked (WWEIA food code 24208510)*	
*4.94% Frankfurter or hot dog, chicken (WWEIA food code 25210310)*	
*6.65% Turkey or chicken sausage, reduced sodium (WWEIA food code 25221855)*	

USDA meat composite details were obtained from the Food Pattern Modeling Report [25]. Proportions of different foods in minimally processed and further processed meat and poultry composites were based on the population-weighted consumptions for NHANES 2017–2018 participants (*n* = 7036; age 2+ years). WWEIA: What We Eat in America; NS: not specified; FDC: Food data central.

**Table 2:** Nutrient profiles per 3 oz (85 g) of meat and poultry composites.

	USDA Meat	Minimally Processed Meat	Minimally Processed Poultry	Further Processed Meat	Further Processed Poultry
**Macronutrients**					
Energy (kcal)	131	151.27	140.99	166.62	186.43
Protein (g)	20.8	24.83	24.93	18.21	16.73
Total fat (g)	4.38	5.17	3.84	8.51	9.68
Carbohydrate (g)	0.84	0.02	0.00	3.37	7.52
Dietary fiber (g)	0.03	0.00	0.00	0.05	0.37
Cholesterol (mg)	60.2	68.89	81.86	51.56	55.55
Saturated fatty acids (g)	1.44	1.96	0.78	2.79	1.81
Monounsaturated fatty acids (g)	1.74	2.23	1.20	3.67	3.60
Polyunsaturated fatty acids (g)	0.42	0.32	0.88	1.09	2.89
**Minerals**					
Calcium (mg)	7.41	11.00	6.60	7.86	10.87
Iron (mg)	1.65	1.54	0.44	0.98	0.54
Magnesium (mg)	18.4	21.91	23.72	19.78	20.19
Phosphorus (mg)	191	215.73	191.60	231.02	202.85
Potassium (mg)	279	345.56	284.54	372.02	257.44
Sodium (mg)	299	364.91	318.69	625.03	461.27
Zinc (mg)	3.45	3.92	0.94	2.21	0.65
Copper (mg)	0.15	0.07	0.04	0.09	0.04
Selenium (µg)	24.1	33.18	25.52	28.52	16.71
**Vitamins**					
Vitamin A, RAE (µg)	41.5	0.59	6.86	2.53	4.58
Vitamin E, ATE (mg)	0.21	0.23	0.63	0.29	0.73
Vitamin D (µg)	0.28	0.16	0.03	0.39	0.11
Vitamin C (mg)	0.09	0.00	0.00	0.06	0.53
Thiamin (mg)	0.18	0.20	0.07	0.26	0.07
Riboflavin (mg)	0.18	0.21	0.18	0.21	0.15
Niacin (mg)	4.89	6.91	8.55	5.49	6.24
Vitamin B_6_ (mg)	0.33	0.55	0.70	0.31	0.32
Vitamin B_12_ (µg)	1.74	1.32	0.23	0.67	0.24
Total choline (mg)	78.0	75.05	64.65	66.28	41.12
Vitamin K (µg)	0.90	0.88	1.28	2.21	3.57
Folate, DFE (µg)	NA	4.72	6.09	6.75	8.86

The nutritional profile of USDA meat was obtained from the Food Pattern Modeling Report [25]. Nutrients profiles for all representative meat and poultry foods (except for ground beef) were obtained from USDA’s Food and Nutrient Database for dietary Studies (FNDDS) 2017–2018 specific for NHANES 2017–2018 [26]. Nutrient profile for ground beef (FDC ID 173113) was obtained using USDA Food Data Central [27]. Nutrient profiles for meat and poultry composites per 3 oz (85 g) were computed by adding the nutrients of component foods in the proportions as presented in Table 1 (for example: nutrient profile for minimally processed meat was computed as 69.30% minimally processed beef (83.85% Beef steak, broiled or baked, lean only eaten + 16.15% Beef steak, fried, lean only eaten) + 30.70% minimally processed pork (Pork chop, broiled or baked, lean only eaten)). ATE: alpha tocopherol equivalents; DFE, dietary folate equivalents; RAE: retinol activity equivalents.

**Table 3:** Energy and nutrients in 2000 kcal Healthy Dietary Patterns before and after removal of a 3 oz (85 g) serving of USDA meat.

	2000 kcal Healthy US-Style Pattern			2000 kcal Healthy Mediterranean Style Pattern		
	Baseline	After Removal of 3 oz (85 g) Serving of USDA Meat	After Isocaloric Removal of 3 oz (85 g) Serving of USDA Meat	Baseline	After Removal of 3 oz (85 g) Serving of USDA Meat	After Isocaloric Removal of 3 oz (85 g) Serving of USDA Meat
**Macronutrients**						
Energy (kcal)	2001	1870	2001	2085	1954	2085
Protein (g)	92	71.2 *	76.2 *	99	78.2 *	83.4 *
Total fat (g)	71	66.6	71.3	72	67.6	72.2
Carbohydrate (g)	259	258	276	271	270	288
Dietary fiber (g)	30	30.0	32.1	31	31.0	33.0
Cholesterol (mg)	214	154 *	165 *	237	177 *	189 *
Saturated fatty acids (g)	18	16.6	17.7	18	16.6	17.7
Monounsaturated fatty acids (g)	25	23.3	24.9	26	24.3	25.9
Polyunsaturated fatty acids (g)	22	21.6	23.1	23	22.6	24.1
**Minerals**						
Calcium (mg)	1278	1271	1360	1297	1290	1376
Iron (mg)	14	12.4 *	13.2	15	13.4 *	14.2
Magnesium (mg)	358	340	363	377	359	383
Phosphorus (mg)	1654	1463 *	1566	1740	1549 *	1653
Potassium (mg)	3390	3111	3329	3628	3349	3574
Sodium (mg)	1658	1359 *	1454 *	1740	1441 *	1538 *
Zinc (mg)	13	9.55 *	10.2 *	13	9.6 *	10.2 *
Copper (mg)	1.4	1.25 *	1.34	1.5	1.35 *	1.44
Selenium (µg)	113	88.9 *	95.1 *	127	103 *	110 *
**Vitamins**						
Vitamin A, RAE (µg)	898	857	917	914	873	931
Vitamin E, ATE (mg)	10	9.79	10.5	11	10.8	11.5
Vitamin D (µg)	7.5	7.22	7.72	9	8.72	9.30
Vitamin C (mg)	129	129	138	145	145	155
Thiamin (mg)	1.8	1.62 *	1.73	1.9	1.72	1.84
Riboflavin (mg)	2	1.82	1.95	2	1.82	1.94
Niacin (mg)	23	18.1 *	19.4 *	25	20.1 *	21.5 *
Vitamin B_6_ (mg)	2.2	1.87 *	2.00	2.3	1.97 *	2.10
Vitamin B_12_ (µg)	6.2	4.46 *	4.77 *	7.3	5.56 *	5.93 *
Total choline (mg)	355	277 *	296 *	378	300 *	320 *
Vitamin K (µg)	140	139	149	142	141	151
Folate, DFE (µg)	513	NA	NA	527	NA	NA

Baseline nutritional profiles of 2000 kcal Heathy Dietary Patterns were obtained from Food Pattern Modeling Report [25]. Nutrient profiles after removal of USDA meat were computed by removing the nutrients of USDA meat (Table 2) from the nutrients of the Healthy Dietary Patterns. Calories were adjusted from the rest of the diet to match the baseline calories in the isocaloric removal of USDA meat. ATE: alpha tocopherol equivalents; DFE, dietary folate equivalents; RAE: retinol activity equivalents. * Indicates ≥10% change from baseline.

**Table 4:** Energy and nutrients in 2000 kcal Healthy Dietary Patterns before and after removal of a 3 oz (85 g) serving of minimally processed meat.

	2000 kcal Healthy US-Style Pattern			2000 kcal Healthy Mediterranean Style Pattern		
	Baseline	After Removal of 3 oz (85 g) Serving of Minimally Processed Meat	After Isocaloric Removal of 3 oz (85 g) Serving of Minimally Processed Meat	Baseline	After Removal of 3 oz (85 g) Serving of Minimally Processed Meat	After Isocaloric Removal of 3 oz (85 g) Serving of Minimally Processed Meat
**Macronutrients**						
Energy (kcal)	2001	1850	2001	2085	1934	2085
Protein (g)	92	67.2 *	72.7 *	99	74.2 *	80 *
Total fat (g)	71	65.8	71.2	72	66.8	72.1
Carbohydrate (g)	259	259	280	271	271	292
Dietary fiber (g)	30	30.0	32.5	31	31.0	33.4
Cholesterol (mg)	214	145 *	157 *	237	168 *	181 *
Saturated fatty acids (g)	18	16.0 *	17.4	18	16.0 *	17.3
Monounsaturated fatty acids (g)	25	22.8	24.6	26	23.8	25.6
Polyunsaturated fatty acids (g)	22	21.7	23.4	23	22.7	24.4
**Minerals**						
Calcium (mg)	1278	1267	1371	1297	1286	1387
Iron (mg)	14	12.5 *	13.5	15	13.5 *	14.5
Magnesium (mg)	358	336	364	377	355	383
Phosphorus (mg)	1654	1438 *	1556	1740	1524 *	1644
Potassium (mg)	3390	3044 *	3293	3628	3282 *	3539
Sodium (mg)	1658	1293 *	1399 *	1740	1375 *	1483 *
Zinc (mg)	13	9.08 *	9.83 *	13	9.08 *	9.79 *
Copper (mg)	1.4	1.33	1.44	1.5	1.43	1.55
Selenium (µg)	113	79.8 *	86.3 *	127	93.8 *	101 *
**Vitamins**						
Vitamin A, RAE (µg)	898	897	971	914	913	985
Vitamin E (ATE) (mg)	10	9.77	10.57	11	10.8	11.6
Vitamin D (µg)	7.5	7.34	7.94	9	8.84	9.53
Vitamin C (mg)	129	129	140	145	145	156
Thiamin (mg)	1.8	1.60 *	1.73	1.9	1.70 *	1.83
Riboflavin (mg)	2	1.79 *	1.94	2	1.79 *	1.93
Niacin (mg)	23	16.1 *	17.4 *	25	18.1 *	19.5 *
Vitamin B_6_ (mg)	2.2	1.65 *	1.78 *	2.3	1.75 *	1.89 *
Vitamin B_12_ (µg)	6.2	4.88 *	5.28 *	7.3	5.98 *	6.45 *
Total choline (mg)	355	280 *	303 *	378	303 *	327 *
Vitamin K (µg)	140	139	150	142	141	152
Folate, DFE (µg)	513	508	550	527	522	563

Baseline nutritional profiles of 2000 kcal Heathy Dietary Patterns were obtained from Food Pattern Modeling Report [25]. Nutrient profiles after the removal of minimally processed meat were computed by subtracting the nutrients of minimally processed meat (Table 2) from the nutrients of the Healthy Dietary Patterns. Calories were adjusted from the rest of the diet to match the baseline calories in the isocaloric removal of minimally processed meat. ATE: alpha tocopherol equivalents; DFE, dietary folate equivalents; RAE: retinol activity equivalents. * Indicates ≥10% change from baseline.

**Table 5:** Energy and nutrients in 2000 kcal Healthy Dietary Patterns before and after removal of a 3 oz (85 g) serving of minimally processed poultry.

	2000 kcal Healthy US-Style Pattern			2000 kcal Healthy Mediterranean Style Pattern		
	Baseline	After Removal of 3 oz (85 g) Serving of Minimally Processed Poultry	After Isocaloric Removal of 3 oz (85 g) Serving of Minimally Processed Poultry	Baseline	After Removal of 3 oz (85 g) Serving of Minimally Processed Poultry	After Isocaloric Removal of 3 oz (85 g) Serving of Minimally Processed Poultry
**Macronutrients**						
Energy (kcal)	2001	1860	2001	2085	1944	2085
Protein (g)	92	67.1 *	72.1 *	99	74.1 *	79.4 *
Total fat (g)	71	67.2	72.3	72	68.2	73.1
Carbohydrate (g)	259	259	279	271	271	291
Dietary fiber (g)	30	30.0	32.3	31	31.0	33.2
Cholesterol (mg)	214	132 *	142 *	237	155 *	166 *
Saturated fatty acids (g)	18	17.2	18.5	18	17.2	18.5
Monounsaturated fatty acids (g)	25	23.8	25.6	26	24.8	26.6
Polyunsaturated fatty acids (g)	22	21.1	22.7	23	22.1	23.7
**Minerals**						
Calcium (mg)	1278	1271	1368	1297	1290	1384
Iron (mg)	14	13.6	14.6	15	14.6	15.6
Magnesium (mg)	358	334	360	377	353	379
Phosphorus (mg)	1654	1462 *	1573	1740	1548 *	1661
Potassium (mg)	3390	3105	3341	3628	3343	3586
Sodium (mg)	1658	1339 *	1441 *	1740	1421 *	1524 *
Zinc (mg)	13	12.1	13	13	12.1	12.9
Copper (mg)	1.4	1.36	1.46	1.5	1.46	1.56
Selenium (µg)	113	87.5 *	94.1 *	127	101 *	109 *
**Vitamins**						
Vitamin A, RAE (µg)	898	891	959	914	907	973
Vitamin E (ATE) (mg)	10	9.37	10.1	11	10.4	11.1
Vitamin D (µg)	7.5	7.47	8.03	9	8.97	9.62
Vitamin C (mg)	129	129	139	145	145	156
Thiamin (mg)	1.8	1.73	1.86	1.9	1.83	1.96
Riboflavin (mg)	2	1.82	1.96	2	1.82	1.95
Niacin (mg)	23	14.4 *	15.5 *	25	16.4 *	17.6 *
Vitamin B_6_ (mg)	2.2	1.50 *	1.62 *	2.3	1.60 *	1.72 *
Vitamin B_12_ (µg)	6.2	5.97	6.42	7.3	7.07	7.58
Total choline (mg)	355	290 *	312 *	378	313 *	336 *
Vitamin K (µg)	140	139	149	142	141	151
Folate, DFE (µg)	513	507	545	527	521	559

Baseline nutritional profiles of 2000 kcal Heathy Dietary Patterns were obtained from Food Pattern Modeling Report [25]. Nutrient profiles after removal of minimally processed poultry were computed by subtracting the nutrients of minimally processed poultry (Table 2) from the nutrients of the Healthy Dietary Patterns. Calories were adjusted from the rest of the diet to match the baseline calories in the isocaloric removal of minimally processed poultry. ATE: alpha tocopherol equivalents; DFE, dietary folate equivalents; RAE: retinol activity equivalents. * Indicates ≥10% change from baseline.

**Table 6:** Energy and nutrients in 2000 kcal Healthy Dietary Pattern before and after removal of a 3 oz (85 g) serving of further processed meat.

	2000 kcal Healthy US-Style Pattern			2000 kcal Healthy Mediterranean Style Pattern		
	Baseline	After Removal of 3 oz (85 g) Serving of Further Processed Meat	After Isocaloric Removal of 3 oz (85 g) Serving of Further Processed Meat	Baseline	After Removal of 3 oz (85 g) Serving of Further Processed Meat	After Isocaloric Removal of 3 oz (85 g) Serving of Further Processed Meat
**Macronutrients**						
Energy (kcal)	2001	1834	2001	2085	1918	2085
Protein (g)	92	73.8 *	80.5 *	99	80.8 *	87.8
Total fat (g)	71	62.5 *	68.2	72	63.5 *	69.0
Carbohydrate (g)	259	256	279	271	268	291
Dietary fiber (g)	30	30.0	32.7	31	31.0	33.6
Cholesterol (mg)	214	162 *	177 *	237	185 *	202 *
Saturated fatty acids (g)	18	15.2 *	16.6	18	15.2 *	16.5
Monounsaturated fatty acids (g)	25	21.3 *	23.3	26	22.3 *	24.3
Polyunsaturated fatty acids (g)	22	20.9	22.8	23	21.9	23.8
**Minerals**						
Calcium (mg)	1278	1270	1386	1297	1289	1401
Iron (mg)	14	13.0	14.2	15	14.0	15.2
Magnesium (mg)	358	338	369	377	357	388
Phosphorus (mg)	1654	1423 *	1552	1740	1509 *	1640
Potassium (mg)	3390	3018 *	3292	3628	3256 *	3539
Sodium (mg)	1658	1033 *	1127 *	1740	1115 *	1212 *
Zinc (mg)	13	10.8 *	11.8	13	10.8 *	11.7 *
Copper (mg)	1.4	1.31	1.43	1.5	1.41	1.54
Selenium (µg)	113	84 *	92 *	127	98 *	107 *
**Vitamins**						
Vitamin A, RAE (µg)	898	895	977	914	911	991
Vitamin E (ATE) (mg)	10	9.71	10.59	11	10.7	11.6
Vitamin D (µg)	7.5	7.11	7.75	9	8.61	9.35
Vitamin C (mg)	129	129	141	145	145	158
Thiamin (mg)	1.8	1.54 *	1.68	1.9	1.64 *	1.78
Riboflavin (mg)	2	1.79 *	1.95	2	1.79 *	1.95
Niacin (mg)	23	17.5 *	19.1 *	25	19.5 *	21.2 *
Vitamin B_6_ (mg)	2.2	1.89 *	2.07	2.3	1.99 *	2.17
Vitamin B_12_ (µg)	6.2	5.53 *	6.03	7.3	6.63	7.20
Total choline (mg)	355	289 *	315 *	378	312 *	339 *
Vitamin K (µg)	140	138	150	142	140	152
Folate, DFE (µg)	513	506	552	527	520	565

Baseline nutritional profiles of 2000 kcal Heathy Dietary Patterns were obtained from Food Pattern Modeling Report [25]. Nutrient profiles after removal of further processed meat were computed by subtracting the nutrients of further processed meat (Table 2) from the nutrients of the Healthy Dietary Patterns. Calories were adjusted from the rest of the diet to match the baseline calories in the isocaloric removal of further processed meat. ATE: alpha tocopherol equivalents; DFE, dietary folate equivalents; RAE: retinol activity equivalents. * Indicates ≥10% change from baseline.

**Table 7:** Energy and nutrients in 2000 kcal Healthy Dietary Pattern before and after removal of a 3 oz (85 g) serving of further processed poultry.

	2000 kcal Healthy US-Style Pattern			2000 kcal Healthy Mediterranean Style Pattern		
	Baseline	After Removal of 3 oz (85 g) Serving of Further Processed Poultry	After Isocaloric Removal of 3 oz (85 g) Serving of Further Processed Poultry	Baseline	After Removal of 3 oz (85 g) Serving of Further Processed Poultry	After Isocaloric Removal of 3 oz (85 g) Serving of Further Processed Poultry
**Macronutrients**						
Energy (kcal)	2001	1815	2001	2085	1899	2085
Protein (g)	92	75.3 *	83 *	99	82.3 *	90.3
Total fat (g)	71	61.3 *	67.6	72	62.3 *	68.4
Carbohydrate (g)	259	251	277	271	263	289
Dietary fiber (g)	30	29.6	32.7	31	30.6	33.6
Cholesterol (mg)	214	158 *	175 *	237	181 *	199 *
Saturated fatty acids (g)	18	16.2 *	17.9	18	16.2 *	17.8
Monounsaturated fatty acids (g)	25	21.4 *	23.6	26	22.4 *	24.6
Polyunsaturated fatty acids (g)	22	19.1 *	21.1	23	20.1 *	22.1
**Minerals**						
Calcium (mg)	1278	1267	1397	1297	1286	1412
Iron (mg)	14	13.5	14.8	15	14.5	15.9
Magnesium (mg)	358	338	373	377	357	392
Phosphorus (mg)	1654	1451 *	1600	1740	1537 *	1688
Potassium (mg)	3390	3133 *	3454	3628	3371	3702
Sodium (mg)	1658	1197 *	1320 *	1740	1279 *	1404 *
Zinc (mg)	13	12.3	13.6	13	12.3	13.6
Copper (mg)	1.4	1.36	1.50	1.5	1.46	1.60
Selenium (µg)	113	96 *	106	127	110 *	121
**Vitamins**						
Vitamin A, RAE (µg)	898	893	985 *	914	909	999
Vitamin E (ATE) (mg)	10	9.27	10.2	11	10.3	11.3
Vitamin D (µg)	7.5	7.39	8.15	9	8.89	9.77
Vitamin C (mg)	129	128	142 *	145	144	159 *
Thiamin (mg)	1.8	1.73	1.91	1.9	1.83	2.01
Riboflavin (mg)	2	1.85	2.03	2	1.85	2.03
Niacin (mg)	23	16.8 *	18.5 *	25	18.8 *	20.6 *
Vitamin B_6_ (mg)	2.2	1.88 *	2.08	2.3	1.98 *	2.18
Vitamin B_12_ (µg)	6.2	5.96	6.57	7.3	7.06	7.75
Total choline (mg)	355	314 *	346	378	337 *	370
Vitamin K (µg)	140	136	150	142	138	152
Folate, DFE (µg)	513	504	556	527	518	569

Baseline nutritional profiles of 2000 kcal Heathy Dietary Patterns were obtained from Food Pattern Modeling Report [25]. Nutrient profiles after removal of further processed poultry were computed by subtracting the nutrients of further processed poultry (Table 2) from the nutrients of the Healthy Dietary Patterns. Calories were adjusted from the rest of the diet to match the baseline calories in the isocaloric removal of further processed poultry. ATE: alpha tocopherol equivalents; DFE, dietary folate equivalents; RAE: retinol activity equivalents. * Indicates ≥10% change from baseline.

## Data Availability

All data obtained for this study are publicly available at: https://www.dietaryguidelines.gov/sites/default/files/2020-07/FoodPatternModeling_Report_2YearsandOlder.pdf; http://www.cdc.gov/nchs/nhanes/; https://fdc.nal.usda.gov/ (accessed on 7 May 2022).
